# Optimising Asthma Self-Management: Preliminary Validation of an Arabic Version of the Inhaler Technique Questionnaire

**DOI:** 10.3390/pharmacy13010006

**Published:** 2025-01-20

**Authors:** Malath Al-Juhaishi, Chiao Xin Lim, Ieva Stupans, Wejdan Shahin, Thilini R. Thrimawithana, Vincent Chan

**Affiliations:** Pharmacy, School of Health and Biomedical Sciences, RMIT University, Melbourne, VIC 3083, Australia; s3540178@student.rmit.edu.au (M.A.-J.); chiao.xin.lim@rmit.edu.au (C.X.L.); ieva.stupans@rmit.edu.au (I.S.); wejdan.shahin@rmit.edu.au (W.S.); thilini.thrimawithana@rmit.edu.au (T.R.T.)

**Keywords:** inhaler technique, metered-dose inhaler (MDI), asthma management, Arabic-speaking populations, respiratory health

## Abstract

Background: Correct inhaler technique is vital for managing respiratory conditions like asthma. Patients from culturally and linguistically diverse backgrounds are at higher risk of sub-optimal adherence and errors in inhaler technique. This study aimed to validate an Arabic version of the inhaler technique questionnaire for self-assessment of the metered-dose inhaler (MDI) technique by assessing agreement between observed and self-reported techniques among Arabic-speaking individuals with asthma in Australia. Methods: Observational assessments of 30 participants using an MDI, followed by completion of the translated inhaler technique questionnaire by the same participants. The questionnaire comprised nine questions pertaining to the inhaler technique. The level of agreement between the observed and self-reported technique for each of the nine MDI technique steps was subsequently determined using intraclass correlation. Results: The majority of participants were women, aged 25–44 years (70%). An overall Kappa score of 0.768 indicated good agreement between observed and self-reported datasets, with stepwise agreement ranging from 52.4–100%. Steps involving taking a big breath before inhaler use (step 2) and exhaling slowly (step 8) were the least well correlated. Conclusions: The preliminary validated MDI inhaler technique questionnaire may be used as a self-assessment tool by Arabic speakers, aiding healthcare professionals, and empowering individuals living with asthma to self-manage their condition.

## 1. Introduction

In the management of asthma, inhaled therapy is crucial, and the standard metered-dose inhaler (MDI) is commonly employed [1,2]. However, incorrect device usage remains a barrier to optimal treatment outcomes [1,2,3]. Incorrect inhaler technique is associated with poor disease outcomes, such as inadequate asthma control and increased exacerbations, significantly impacting the health-economic burden for patients and the healthcare system [4,5]. Approximately 2% of Australia’s population was born in the Middle East or North Africa, with Arabic being one of the top three non-English languages spoken at home by around 1.4% of the population [6,7]. A previous study on Arabic-speaking immigrants’ asthma management in Australia with limited English proficiency revealed treatment gaps such as poor asthma control, non-adherence, and low awareness of self-management skills [8].

The conventional approach for assessing the inhaler technique involves direct patient observation [3,9,10]. However, direct observation may be infrequent due to factors such as a lack of awareness among healthcare providers regarding the significance of routine technique reassessment, time constraints during clinic visits, and a limited understanding among healthcare professionals responsible for patient education on the crucial factors influencing optimal inhaled medication delivery [11]. Moreover, variabilities in inhaler technique evaluations by healthcare professionals further complicate the process by confusing the patients. An alternative to direct observation is patient self-assessment through a questionnaire, offering advantages such as time efficiency, and the elimination of inter-evaluator differences common in face-to-face observations. In addition, this approach may enhance patient engagement in health care.

Numerous tools used to assess MDI techniques have been primarily developed in English, with few being translated and validated in languages other than English [9,12,13]. However, to the author’s knowledge, there are currently no validated tools available in the Arabic language for evaluating MDI techniques. Hence, there is a critical need for a reliable measure of MDI techniques that is validated for use by Arabic-speaking patients [13].

The aim of this study is to develop and validate an Arabic questionnaire designed for self-assessment of MDI techniques, by evaluating the agreement between observed and self-reported techniques among Arabic-speaking individuals with asthma residing in Australia.

## 2. Materials and Methods

### 2.1. Inhaler Technique Evaluation Tool

Two questionnaires, the Inhaler Technique Questionnaire developed in 1998 and the Inhaler Technique Questionnaire (InTeQ) developed in 2022 [12,13], were considered for this study to assess MDI inhaler techniques. Both questionnaires are validated and have been successfully employed in prior studies [14]. The authors made the decision to initially provide both questionnaires to participants to investigate participant preferences towards the questionnaires.

Both questionnaires underwent a meticulous translation process to ensure accuracy for Arabic-speaking participants. One researcher translated both questionnaires into Arabic, and another bilingual researcher conducted a back translation to validate the accuracy of the translation. Following this, both questionnaires were pilot-tested with five Arabic-speaking participants, all of whom preferred the Inhaler Technique Questionnaire [13]. The pilot testing also ensured the cultural appropriateness of the questionnaire content. Feedback from pilot testing was incorporated to refine the wording and ensure cultural relevance. The Inhaler Technique Questionnaire was then professionally translated into Arabic by a certified translator. Subsequently, two bilingual researchers translated the Arabic version back into English, and this translation was compared to the original English version to verify the meaning of each question. For the translation, we used Modern Standard Arabic (MSA), which is widely understood across Arabic-speaking populations. While regional dialects have variations, MSA serves as a neutral and universal medium for written communication, minimising dialect-related biases.

The tool comprises nine steps that cover essential aspects of inhaler use, including breathing techniques, breath-holding duration, and waiting periods between inhalations.

### 2.2. Study Design and Setting

After obtaining approval from the University Ethics Committee (25419), data collection took place between September 2022 and January 2024. A convenience sampling method was employed to recruit participants from two Australian Arabic immigrant Facebook groups which served as a platform for social interaction and support among Middle Eastern refugees and migrants. Eligibility criteria include: (1) adults aged 18 years and above, diagnosed with asthma; (2) migrated to Australia from one of the 19 Middle Eastern countries [15]; and (3) use medication delivered through an MDI. Exclusions were made for: (1) individuals relying exclusively on spacer devices alongside their MDI; (2) employed a non-conventional MDI device, such as breath actuated inhaler; or (3) unable to speak and read Arabic. Eligible participants received a participant information sheet, a comprehensive study explanation, and informed consent was obtained. All participants received a AUD 15 voucher as an honorarium.

The researcher and the participant mutually agreed upon a suitable time for conducting online video conferencing to facilitate direct observation of the participant’s inhaler technique. The participant’s inhaler technique was assessed and scored by the researcher, each step being aligned to the English version of the Inhaler Technique Questionnaire [13].

One researcher undertook all observations of the participants’ inhaler technique. Four weeks after the initial interview, a follow-up online questionnaire comprising nine questions was distributed to the participants, whereby participants self-rated whether they completed the required steps using the translated Arabic version of the Inhaler Technique Questionnaire.

### 2.3. Data Analysis

Responses for each MDI technique step [13] were categorized as either correct or incorrect and the total MDI technique score was calculated, indicating the number of steps performed correctly (ranging from zero to nine) for each participant.

To ensure the reliability of the observational component, an inter-rater-reliability test was conducted. The interviews were conducted by one author while the other authors independently scored the observed steps. Then, the scores were compared to determine consistency. Discrepancies were resolved through discussion to ensure accuracy.

The level of agreement between the observed and self-reported technique for each of the nine MDI technique steps was subsequently determined using SPSS Software 29.0^®^ through the intraclass correlation measure. A Kappa score above 0.7 was considered a good correlation.

## 3. Results

Thirty Arabic immigrants with asthma participated, mostly women aged 25 to 44. The majority had asthma for less than 15 years (Table 1). The median length of inhaler use was 6 years (IQR 4–14). The agreement between observational and self-reported responses was measured by Kappa coefficients. Among the nine steps (Table 2), step 5 achieved perfect agreement (Kappa = 1). Steps 4, 6, 7, and 9 showed strong agreement (0.8–0.9), while steps 1, 2, 3, and 8 exhibited moderate agreement (0.5–0.6). The overall Kappa score of 0.768 indicates good agreement between datasets, with stepwise agreement ranging from 52.4% to 100%.

Inter-rater reliability testing was initially conducted on ten assessments, with both assessors achieving complete agreement. However, for the remaining 20 participants, inter-rater reliability testing was not performed, as these participants declined to be recorded, and their preferences were respected.

## 4. Discussion

To the best of our knowledge, this is the first study describing the validation of a questionnaire in Arabic for self-reporting of MDI techniques. Using this questionnaire, it was possible to estimate the accuracy of the inhaler technique without directly observing the participants.

This study demonstrated an overall agreement of 76.8% between the observational and self-reported steps, consistent with the findings from a previous study by Erickson et al., who reported an overall agreement of 76.8% [13] using the English version of the Inhaler Technique Questionnaire. This Arabic questionnaire presents good validity in assessing MDI techniques for Arabic-speaking populations, thus empowering Arabic-speaking patients to self-assess their inhaler use. Given the importance of Arabic as one of the top five languages spoken at home worldwide, with 319 million people speaking Arabic [16], this is a significant contribution to improving the management of asthma and quality of life in this population group. This questionnaire tool can prompt patients who find that they have poor inhaler technique to seek assistance from their healthcare providers, thereby promoting active engagement and enhancing patient-centred care.

Our study showed moderate agreement between patient self-reported and observed inhalation techniques, particularly in the steps involving taking a big breath before using the inhaler (step 2) and exhaling slowly (step 8). This discrepancy may be attributed to the written language of these instructions, suggesting a need for modification to enhance clarity and improve patient adherence.

As a result, healthcare professionals should focus on regular, step-by-step assessments of inhaler techniques, particularly addressing these frequent MDI use errors, rather than solely relying on patients’ overall confidence and theoretical knowledge.

Inter-rater reliability was established in this study to enhance the credibility and strength of the collected observational data; test–retest reliability was not undertaken given the limitations of the potential “testing effect” and the small sample size [17]. A larger sample size would also further increase the validity of the results. It is also important to note that this study was conducted with Arabic-speaking immigrants in Australia, which may limit its generalizability to Arabic-speaking populations in other contexts. Previous research has examined the reliability of the English version [14,18]. However, it is important to note that language and cultural differences can impact reliability differently. Additionally, this study focused primarily on criterion validity but did not explore construct validity, and did not assess the internal consistency of the questionnaire items. Furthermore, the participant’s education level was not captured in this study to identify any associations in that context.

Another key limitation is the reliance on questionnaire methodology for data collection, which has specific drawbacks relevant to validating the Arabic version of the inhaler technique questionnaire in patients with asthma [19]. Questionnaire responses may be influenced by social desirability bias, where participants may overstate their proficiency to align with perceived societal norms, affecting the accuracy of inhaler technique responses. Additionally, self-reported data may have inaccuracies due to memory lapses or perceptual biases, impacting the reliability of responses [19]. The cross-sectional nature of most questionnaires limits the ability to establish causation or observe changes over time, which is crucial for comprehensively assessing the effectiveness of the Arabic questionnaire in capturing changes in inhaler technique proficiency among patients with asthma [19].

## 5. Conclusions

This study provided some insights on a preliminary validation of the Arabic version of the inhaler technique questionnaire for evaluating MDI use by Arabic-speaking populations. This tool can assist healthcare professionals in promptly assessing patients’ inhaler techniques across diverse settings including telehealth. It also enables individuals to self-assess their inhaler technique, fostering self-management practice. While not a substitute for direct observation, the questionnaire serves as an effective patient education tool in clinical settings, reinforcing proper MDI techniques and potentially enhancing patient outcomes.

## Figures and Tables

**Table 1:** Participants’ demographics (n = 30).

Characteristics		N
Gender	Female	21
	Male	9
Age (years)	18–24	3
	25–44	21
	45–65	6
Length of diagnosis (years)	≤5	11
	6–14	12
	≥15	6
	Unknown	1

**Table 2:** Intra-class agreement between observed technique and self-reported technique.

Steps	Measure of Agreement Kappa
Step 1: Shake inhaler	0.615
Step 2: Take a big breath in and out before using the inhaler	0.556
Step 3: Keep head straight	0.636
Step 4: Hold the inhaler upright	0.866
Step 5: Put the inhaler inside the mouth	1
Step 6: Begin to breathe in, then push down the canister and continue to breathe in	0.866
Step 7: Hold your breath for about 5 to 10 s before exhaling	0.867
Step 8: Exhale slowly	0.524
Step 9: Push down on the canister 1 time, then wait about a minute before doing it again	0.923
Overall inhaler technique	0.768

Kappa score above 0.5 = moderate agreement; Kappa score above 0.7 = good agreement; Kappa score above 0.8 = strong agreement.

## Data Availability

The datasets generated during and/or analysed during the current study are not publicly available due to ethical reasons. Upon reasonable request, study authors will provide access to data.
